# Economic evaluation of HIV testing options for low-prevalence high-income countries: a systematic review

**DOI:** 10.1186/s13561-021-00318-y

**Published:** 2021-06-07

**Authors:** Olanrewaju Medu, Adegboyega Lawal, Doug Coyle, Kevin Pottie

**Affiliations:** 1 Saskatchewan Health Authority , Regina, SK Canada; 2 University of Saskatchewan, Saskatoon, Canada; 3 University of Ottawa School of Epidemiology and Public Health, Ottawa, Canada; 4 Bruyère Research Institute, Ottawa, Canada

**Keywords:** HIV testing, Economic evaluation, High-income countries

## Abstract

**Introduction:**

This study reviewed the economic evidence of rapid HIV testing versus conventional HIV testing in low-prevalence high-income countries; evaluated the methodological quality of existing economic evaluations of HIV testing studies; and made recommendations on future economic evaluation directions of HIV testing approaches.

**Methods:**

A systematic search of selected databases for relevant English language studies published between Jan 1, 2001, and Jan 30, 2019, was conducted. The methodological design quality was assessed using the Consolidated Health Economic Evaluation Reporting Standards (CHEERS) and the Drummond tool. We reported the systematic review according to the PRISMA guidelines.

**Results:**

Five economic evaluations met the eligibility criteria but varied in comparators, evaluation type, perspective, and design. The methodologic quality of the included studies ranged from medium to high. We found evidence to support the cost-effectiveness of rapid HIV testing approaches in low-prevalence high-income countries. Rapid HIV testing was associated with cost per adjusted life year (QALY), ranging from $42,768 to $90,498. Additionally, regardless of HIV prevalence, rapid HIV testing approaches were the most cost-effective option.

**Conclusions:**

There is evidence for the cost-effectiveness of rapid HIV testing, including the use of saliva-based testing compared to usual care or hospital-based serum testing. Further studies are needed to draw evidence on the relative cost-effectiveness of the distinct options and contexts of rapid HIV testing.

## Introduction

Human immunodeficiency virus (HIV) infection is an important contributor to disease’s global burden and a leading cause of death [1]. With the advent of antiretrovirals and treatment regimens, the disease, when diagnosed early, can be managed. As a result, HIV patients now have an improved quality of life and comparable life expectancies with persons uninfected with HIV [2]. The process of achieving this improved quality of life can be represented by the internationally recognized framework known as the cascade or continuum of care [2, 3]. This framework begins with disease diagnosis through HIV testing, linkage to care, retention in treatment programs, maintenance of treatment adherence and finally sustained viral suppression [4, 5].

HIV testing, early diagnosis and effective treatment improve outcomes significantly for infected individuals and the communities they live [6, 7]. This premise forms the foundation for the highly effective “*treatment as prevention”* approach [2, 8]. Getting people aware of their HIV status has been the focus of HIV control agencies. However, recent UNAIDS data shows that about 50% of people living with HIV are unaware of their diagnosis; for example, Canada, France, Spain and the United States report a substantial proportion of undiagnosed HIV cases [9–13]. Rates of undiagnosed HIV tend to be higher among men who have sex with men, youth and minority population groups [11].

There are currently several HIV testing approaches, including serum and saliva-based screening tests [14–19]. Serum-based testing can be categorized based on the duration to receipt of the test result. In conventional HIV testing, the serum-based results are usually available within a week; however, this may require the client to return to the facility to receive the result. In contrast, rapid testing approaches provide results within 24 h and do not require clients to return for results notification.

There have been clinical effectiveness studies of the various HIV testing approaches; however, economic evaluations of the different approaches from non-American perspectives are lacking. Economic studies have been conducted in high prevalence low-income African countries [20] and in high prevalence communities in the United States and Europe [21–25]. Individual studies and systematic reviews considered the effectiveness of rapid HIV testing [17, 26–28] and cost-effectiveness studies of rapid HIV testing options [29]. Still, there is no review focused on rapid HIV testing’s economic evidence compared to conventional HIV testing in low-prevalence, high-income countries. We focus on these countries because they tend to have similar epidemiology and comparable health care systems.

We seek to address this specific HIV testing evidence gap given the potential to increase access to HIV care and treatment programs to following the United Nations 90–90-90 goals. In particular, the first goal seeks to have 90% of all people living with HIV know their HIV status by 2020 [1]. This systematic review focuses on North America, Australia and Western Europe, areas with low HIV prevalence and high incomes with similar HIV epidemiology. These jurisdictions would benefit from economic evaluation of HIV testing to make informed decisions about cost-effective HIV screening programming and judicious use of health care resources.

The specific aims of this systematic review are to (i) search, select, appraise and synthesize published economic evaluations of HIV testing options; (ii) evaluate the methodological quality of the economic evaluations of HIV testing; (iii) make recommendations regarding future directions for economic evaluation of HIV testing approaches. This review also focuses on the strength and quality of evidence addressing the cost-effectiveness of rapid HIV testing approaches versus conventional testing approaches in managing HIV infection.

## Methods

This work is a systematic review of available literature on any rapid HIV testing approach’s economic evaluations versus conventional serum-based HIV testing. We followed the Preferred Reporting Items for Systematic Reviews and Meta-Analyses (PRISMA) statement in this article’s reporting [30].

### Search strategy/process

We searched the medical literature in Medline (indexed, in-process and other non-), Embase, NHS EED and Tufts Cost-effectiveness analysis (CEA) registry to retrieve all relevant literature. Text words used in the search include ‘economic evaluation’, ‘cost’, ‘cost-effectiveness’, ‘cost-benefit’ or ‘cost-utility’, ‘rapid HIV testing, and ‘HIV testing’. Due to the review team’s limited language competency/expertise and resources, only English language studies were included in the search strategy. The review team also considered studies conducted between Jan 1, 2001, and Jan 30, 2019, in North America, Australia or Western Europe.

### Inclusion criteria

For this review, the inclusion criteria were as follows: an economic evaluation study design that was either an economic evaluation, a clinical trial or model-based evaluation conducted in North America, Australia or Western Europe, involving adult patients aged 16 years and older tested for HIV using at least two of the following four HIV testing approaches (i) whole blood/serum-based hospital-based testing (also referred to as conventional HIV testing approaches); (ii) rapid hospital-based testing; (iii) rapid location-based testing; and (iv) rapid mobile testing.

This review excluded saliva-based testing due to concerns about test performance. Specifically, saliva-based testing options were associated with lower specificity when self-administered than healthcare provider administered tests [31, 32].

Rapid HIV testing has the following three components: (i) voluntary enrolment, (ii) rapid testing with results available within 24 h and (iii) provision of counselling at the delivery of results and treatment options.

The economic evaluations considered in this review included cost-effectiveness, cost-utility or cost-benefit analysis. These would include any of the following outcomes: (i) cost per quality-adjusted life years (QALY); (ii) cost per HIV test; (iii) cost per HIV transmission prevented; or (iv) total cost of HIV testing program. We excluded studies if they considered only one testing approach with no comparator. Additionally, we excluded cost minimization studies because these are not formal economic evaluations and usually are costing exercises where there is no difference in the effect of the comparators [33–35].

Finally, due to the difference in HIV epidemiology and characteristics of the health care systems, economic evaluations from Africa, Eastern Europe and Asia were excluded from this review.

### Data abstraction

Two independent reviewers (OM and AL) selected eligible publications initially based on titles and abstracts. Potentially relevant articles were abstracted using standardized data abstraction form. Disagreement between reviewers was resolved by a third reviewer (DC).

Descriptive data was collected for each economic evaluation, including study objectives, perspective, analysis type and study design, sample size and population studied. Other considerations include comparator(s), intervention, results, and conclusions. Costs have been adjusted to reflect 2018 United States dollars using international exchange rates and the United States Bureau of Labour Statistics inflation calculator for medical costs [36].

### Quality assessment

The methodological quality of HIV testing economic evaluations was assessed using two tools: Drummond’s ten-point criteria for economic evaluations and the 24-item Consolidated Health Economic Evaluation Reporting Standards (CHEERS) checklist [37–39]. For both lists, each item was scored as ‘Yes’ (met the quality criterion), or ‘No’ (did not meet the quality criterion), or ‘Can’t tell’ where there was insufficient evidence to make a decision. A numeric score was not calculated for each study.

For the CHEERS criteria, the “Yes” responses were weighed against the total number of criteria for a percentage. This approach has been used in recently published systematic reviews of economic evaluations [40–43].

The two checklists used had slightly different focus but were nonetheless complimentary. While the Drummond checklist assesses appropriate methodology in the economic evaluation and evaluates the results’ validity, the CHEERS checklist focuses on reporting issues. Using the CHEERS checklist, studies were assessed into three categories: high if they satisfied greater than 75% of the criteria, average (50–75%) and low quality when less than 50% of the criteria was satisfied.

## Results

### Literature search and screening

**Fig. 1:**
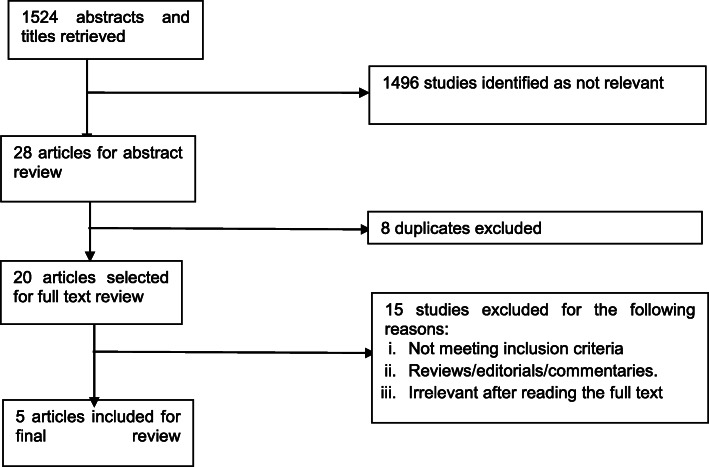
PRISMA Flow Diagram

The initial search resulted in 1524 records. Five studies met our inclusion criteria for this systematic review (See Fig. 1). Studies were excluded because of a lack of comparators in the economic evaluation resulting in the studies being categorized as costing studies. Other reasons for exclusion included studies conducted in jurisdictions outside of specified geographic location, evaluations of hospitals or organization-specific testing programs that were not explicitly evaluations of HIV testing approaches.

### Study and patient characteristics

Five primary articles met the inclusion criteria and were considered for data analysis and synthesis [44–48]. An overview of the study and methodological characteristics, study populations, interventions, and outcomes of the five economic evaluations included in the review are provided in Table 1. The earliest economic evaluations in this review were published in 2005 [47, 49], and the remaining three were published between 2010 and 2012 [44–46]. All the included studies in this review were conducted in the United States.

**Table 1:** Overview of the five economic evaluations reviewed by study characteristics and outcomes with all costs in 2018 US

Authors	Country	Setting	Perspective	Analysis type	Study design (follow up)	Time horizon	Population	Interventions	Outcomes
Cipriano LE et al., 2012 [44]	United States	US Urban center	Societal^a^	Cost-utility	Deterministic dynamic compartmental model	20 years	IDU’s and non-IDU’s in opioid replacement therapy	One-time and repeat interval screening	ICER; Costs per life year; $36,081 per QALY versus one-time screening
Sanders GD et al., 2010 [46]	United States	US	Perfect insurer^b^	Cost-effectiveness	Markov model	Lifetime	Emergency department	Model A: traditional HIV counseling and testing; Model B: nurse-initiated routine screening with traditional HIV testing and counseling; Model C: nurse-initiated routine screening with rapid HIV testing and streamlined counseling	Cost per QALY vs Model A: Model B: Extended dominance Model C: $ 42,769 /QALY; Cost per life year (LY) vs Model A Model B: Extended dominance Model C: $ 31,392.35 /LY
Dowdy DW et al., 2011 [45]	United States	Emergency departments	Societal^a^	Cost-effectiveness	Decision analysis	Lifetime	Persons at higher risk of HIV	Targeted ED HIV screening versus clinic-based approaches	$ 96,727.44 for targeted screening program; $ 53.51 per screening test; $ 90,498.34 /QALY for targeted HIV screening versus clinic-based approaches
Paltiel A D et al., 2005 [47]	United States	USA	Societal^a^	Cost-utility	Model-based evaluation: Monte Carlo, state-transition framework	Lifetime	High risk, CDC threshold and US general cohort	Routine voluntary HIVCTR; Testing at presentation with opportunistic infections	High risk population: One-time ELISA versus current practice: $ 51,283.93 /QALY, More frequent screening >$50,000/QALY In the general population: all screening regiments are >$50,000/QALY
Walensky R P et al., 2005 [48, 49]	United States	Hypothetical cohort of 100 million US inpatients	Societal^a^	Cost-utility	State-transition simulation model	Lifetime	Hypothetical cohort of 100 million US inpatients	HIV screening based on HIV prevalence	Screening versus no screening, $ 50,429.20 /QALY in settings with 1% HIV prevalence; $ 91,171.44 in settings with 0.1% HIV prevalence

^a^Societal perspective refers to consideration of all foreseeable benefits/effects (positive and negative outcomes) including those outside of the healthcare system

^b^The perfect insurer refers to considerations of costs to the insurer and patient, and corresponds to what most studies term a societal perspective

All the included studies were model-based economic evaluations comprised of two cost-effectiveness studies [45, 46] and three cost-utility studies [44, 47, 48].

Sanders et al. [46] was conducted from the healthcare insurer’s perspective, and the remaining four [44, 45, 47, 48] were from a societal perspective. Four studies [45–48] considered a lifetime horizon in the evaluation, and one study [32] considered a 20-year time horizon.

All studies included populations considered at high risk for HIV (prevalence higher than 1%), such as injection drug users and inner-city US populations as well as members of the general population with assumed prevalence higher than 1%, while the general population prevalence was approximately 0.1% [44–48].

### Comparative interventions

Comparisons considered in the included studies include one-time and repeat interval rapid screening, rapid emergency department (ED) testing versus usual care, various rapid testing approaches [44–46, 48]. While varied, these approaches have the common theme of a rapid HIV testing arm compared to the usual standard of care or other rapid HIV testing approaches.

All five studies reported outcomes as cost per quality-adjusted life-years and found that rapid HIV testing approaches were cost-effective. Sanders et al., [46] found nurse-initiated routine screening with rapid HIV testing and streamlined counselling was more cost-effective at $42,769/QALY, while Dowdy et al., [45] found targeted HIV screening in emergency departments cost-effective at $90,498.34 per QALY. Furthermore, at a willingness to pay a threshold of $100,000 per QALY gained, this option was cost-effective in 89% of simulated scenarios [45, 46].

The comparisons considered in the included studies include one-time and repeat interval rapid screening [44] and various rapid testing approaches, including oral testing [45–47].

When varying prevalence of HIV was considered, rapid HIV screening was found to range in cost-effectiveness from $50,429/QALY in settings with at least 1% HIV prevalence to $91,171/QALY in settings with 0.1% HIV prevalence [48]. Another study by Paltiel et al., [47] found that one-time screening costs $51,284/QALY among high-risk populations. The study further found that testing every 5 years costs $71,227/QALY, and by reducing the frequency of testing to every 3 years, it cost $89,746/QALY [47].

### Study quality assessment

Table 2 presents the distribution of scores across each of the 10-item Drummond checklist according to whether or not each study fulfilled the criterion (or was not applicable) in terms of study design, execution and reporting of relevant information on methods used in the study [50]. Across included studies, we noticed variability in methodological quality.

**Table 2:** Summary of Drummond evaluation of methodological quality

Drummond criteria	Cipriano LE et al., 2012 [44]	Sanders GD et al., 2010 [46]	Dowdy DW et al., 2011 [45]	Paltiel AD et al., 2005 [47]	Walensky RP et al., 2005 [48, 49]
Well-defined question?	Yes	Yes	Yes	Can’t tell	Can’t tell
Adequate description of comparators?	Yes	Yes	Can’t tell	Can’t tell	Can’t tell
Evidence of effectiveness?	Yes	Yes	Yes	Yes	Yes
Relevant costs/consequences?	Yes	Yes	Yes	Can’t tell	Can’t tell
Costs/consequences accurately measured?	No	No	Can’t tell	Can’t tell	Can’t tell
Were the valuation costs/consequences credible?	Yes	Can’t tell	Can’t tell	Can’t tell	Can’t tell
Was discounting used as appropriate?	Yes	Yes	Yes	Yes	Yes
Were incremental analyses appropriately reported?	Yes	Yes	Yes	Yes	Yes
Were sensitivity analyses reported?	Yes	Yes	Yes	Yes	Yes
Was the discussion adequate?	Yes	Yes	Yes	Yes	Yes
Percent of criteria met	90%	80%	70%	50%	50%

Specific concerns on the Drummond checklist showed that most studies did not accurately measure the costs and consequences or justify that the valuation costs and consequences were credible (See Table 2). Paltiel [47] and Walensky [48] did not adequately report five of the ten criteria identified on the Drummond checklist. These studies were considered lower quality relative to the other studies.

We present a summary of the included studies’ quality evaluation based on the CHEERS checklist in Table 3. Based on this assessment, all included studies were assessed as high quality.

**Table 3:** Quality of the included studies based on the CHEERS checklist

Study	Q1	Q2	Q3	Q4	Q5	Q6	Q7	Q8	Q9	Q10	Q11	Q12	Q13	Q14	Q15	Q16	Q17	Q18	Q19	Q20	Q21	Q22	Q23	Q24	Score
Cipriano LE et al., 2012 [44]	Yes	No	Yes	Yes	Yes	Yes	Yes	Yes	Yes	Yes	Yes	No	No	Yes	Yes	Yes	No	Yes	Yes	No	Yes	Yes	Yes	Yes	79%
Sanders GD et al., 2010 [46]	Yes	No	Yes	Yes	Yes	Yes	Yes	Yes	Yes	Yes	NA	No	Yes	Yes	Yes	Yes	No	Yes	Yes	Yes	Yes	Yes	Yes	Yes	83%
Dowdy DW et al., 2011 [45]	Yes	Yes	Yes	Yes	Yes	Yes	Yes	Yes	Yes	Yes	Yes	No	Yes	Yes	Yes	No	No	Yes	Yes	Yes	Yes	Yes	Yes	Yes	88%
Paltiel A D et al., 2005 [47]	Yes	No	Yes	Yes	Yes	Yes	Yes	Yes	Yes	No	Yes	No	Yes	Yes	Yes	Yes	Yes	No	Yes	Yes	Yes	Yes	Yes	Yes	83%
Walensky R P et al., 2005 [48, 49]	Yes	No	Yes	Yes	Yes	Yes	Yes	Yes	Yes	Yes	Yes	No	Yes	Yes	Yes	Yes	Yes	No	Yes	Yes	Yes	Yes	Yes	Yes	88%
Completed criteria	100%	20%	100%	100%	100%	100%	100%	100%	100%	80%	80%	0%	80%	100%	100%	80%	40%	60%	100%	80%	100%	100%	100%	100%	

Our review found that most criteria were adequately reported. We note that some criteria were generally underreported. For example, the abstract usually did not have enough information about the base case and the outcome’s uncertainty. We also identify that none of the studies adequately reported the populations and methods used to elicit outcome preferences. There were also concerns about how the studies reported the analytic methods used in the evaluations.

All included studies [44–48] performed a sensitivity analysis and provided varying depths of reporting about the sensitivity designs used (Table 4). Sanders et al. [46] conducted a probabilistic sensitivity analysis (PSA), Paltiel et al. [47] conducted both univariate and multivariate deterministic analysis. The remaining three evaluations conducted only univariate deterministic sensitivity analysis [44, 45, 48].

**Table 4:** Sensitivity analysis descriptions

Authors	Analysis type	Study design (follow up)	Modelling method	Type of sensitivity analysis	Parameters
Cipriano LE et al., 2012 [44]	Cost-utility	Deterministic dynamic compartmental model	Dynamic compartmental model	Univariate deterministic sensitivity analysis	No of IDU’s by city; Prevalence of HIV and HCV
Sanders GD et al., 2010 [46]	Cost-effectiveness	Trial based Markov model	Markov model	Univariate and multivariate deterministic sensitivity analysis; Probabilistic sensitivity analysis	HIV test characteristics; Test probability; Probability of undiagnosed HIV; Probabilities of receiving HIV test result given positive and negative result
Dowdy DW et al., 2011 [45]	Cost-effectiveness	Decision analysis	Decision analysis	Univariate deterministic sensitivity analysis	Prevalence of undiagnosed HIV; Annual HIV transmission rate; Lifetime cost of treating new HIV cases; Monthly test volume; HIV awareness
Paltiel A D et al., 2005 [47]	Cost-utility	Model-based evaluation: Monte Carlo, state-transition framework	State-transition simulation model	Multivariate deterministic sensitivity analysis	Testing frequency; Proportion of persons returning for results; Efficacy of antiretroviral (ARV); Proportion of infected on ARV
Walensky R P et al., 2005 [48, 49]	Cost-utility	State-transition simulation model	State-transition simulation model	Univariate deterministic sensitivity analysis	Testing costs; CD4 counts; HIV Prevalence; Cost of ARV

Table 4 shows the modelling approaches used by the various evaluations included in the analysis. One study [45] was a decision analysis model, three studies [46–48] were transition simulation models, and the last evaluation used a dynamic compartmental model [44]. Two [47, 48] of the transition model evaluations used four-state transition using Cost-Effectiveness of Preventing AIDS Complications (CEPAC) data [51]. The last model-based study used a seven-state model [46].

## Discussion

Economic analysis is imperative to assist with rational decision-making about the allocation of limited health resources. It seeks to provide information about the value of competing health interventions. Our review found five studies that reported economic evaluations of rapid HIV testing approaches in North America. While our inclusion criteria expanded to studies conducted in Western Europe and Australia, we could not find any such evaluations from these countries. For this review, we have adjusted all cost figures to 2018 United States dollars. Economic evaluations reported a wide variation in comparators’ use, evaluation type, perspective, and design; thus, the estimates’ statistical pooling was not feasible.

The studies included in this analysis show that rapid HIV testing approaches, including saliva-based screening tests, may be a cost-effective option compared to usual care or hospital-based serum testing options. The conclusion from the highest methodological quality studies (studies that satisfied most of the conditions identified on the Drummond checklist) showed that rapid testing approaches were cost-effective compared to conventional hospital-based serum HIV testing with an ICER between $36,081 per QALY and $39,376 per QALY [44].

Lower-quality studies also showed that rapid HIV testing approaches were cost-effective at an estimated $51,284 per QALY. We found an increase in the cost per QALY when the test was used in populations with a lower prevalence of HIV increasing to about $91,171 per QALY [47].

Our study considered economic evaluations from high-income countries in North America, Europe, and Australia, where disease pattern is similar. We did not find studies from any country other than the United States; therefore, the estimates provided reflect more of the American health care system. While these ICERs are for the most above the $50,000 per QALY threshold, they are below the higher limits of the $100,000 threshold considered acceptable in some higher-income countries [52–57].

These higher ICER thresholds may potentially reflect an overestimation of the ICER because of the nature of the healthcare system that has shown a trend towards a willingness to pay threshold of US$ 150,000 per QALY gained [58]. These ICER values would be considered acceptable in most of the target countries and jurisdictions. In Canada, for example, the ICER values would be considered acceptable because they are below the maximum of the commonly used Canadian threshold of between $20,000 - $100,000 per QALY gained. In some circumstances, when considering high prevalence populations, the ICER value is lower than $50,000 per QALY gained threshold [34, 53]. While Australia and the United Kingdom do not have a fixed threshold value for ICER given the recommended ICER thresholds, some of the ICER amounts would be considered not acceptable [52, 57].

Two of our included studies [47, 48] used the Cost-Effectiveness of Preventing AIDS Complications (CEPAC) model [51], a mathematical simulation of the detection, natural history and treatment of HIV disease in the US and it is thus expected that the findings would be consistent. With the variations in the settings and populations of interest, rapid HIV testing approaches remained cost-effective compared to conventional approaches.

Most of the studies considered rapid HIV testing as an approach resulting in early detection of disease with subsequent connection to care and treatment shown to result in improved outcomes and the prevention of new cases of HIV. The outcome measures included cost per QALY gained, cost per HIV test and cost per test notification. These outcomes are important because early HIV diagnosis benefits extend beyond potential immediate improvements to individual client health outcomes. Beyond the individual, it includes other considerations such as preventing new HIV transmission. These outcomes are however not adequately reported.

Finally, it is essential to highlight that none of the included studies explicitly considered equity factors, including place of residence, race/ethnicity/culture/language, occupation, gender/sex, religion, education, socioeconomic status, and social capital [59]. The non-inclusion of equity factors is likely because included studies considered the traditional approach of economic evaluation that *‘a QALY is a QALY’* that assumes all outcomes should be weighted equally, regardless of the characteristics of people receiving [33]. There is a school of thought that considers this a value judgment that is questionable when applied to public health and suggest that equity considerations should be incorporated in economic evaluations [60–62].

### Limitations

We identified a few limitations. All studies were conducted in the United States, and published between 2005 and 2012. None of the studies was published since the recent HIV programming management changes, such as the 90–90-90 strategy [1]. The 90–90-90 strategy requires a scaling up of HIV testing and treatment. It aims to have 90% of persons HIV infected tested and aware of their status, 90% of infected persons on antiretroviral treatment and viral suppression in 90% of persons on antiretroviral drugs [1]. Second, we did not find any studies from other high-income economies with similar HIV prevalence. Generalizing these high-income countries may be difficult. Also, this review did not include studies published in languages other than English.

None of the studies considered the cost of rapid HIV testing per new HIV transmission prevented, an outcome that would be significant in advancing an economic argument for the use of rapid HIV testing approaches as an integral population strategy in HIV programming.

In this review, we also note that only one study conducted a probabilistic sensitivity analysis that is considered the only approach that can potentially address uncertainties in all inputs rather than confining this to a subset as is usual in the univariate and multivariate deterministic analysis [63].

The Consumer Price Index (CPI) used to adjust the costs indicates changes in population consumer prices. The CPI is obtained by comparing the cost of a fixed basket of goods and services purchased by consumers [64–66]. This approach is limited because it does not account for other options that may not be included in the fixed basket used in the assessment and likely ignore the cost savings from less costly alternatives [66].

While appropriate for assessing study inclusion criteria, we also identify that the Drummond checklist may not adequately address contextual and health system factors related to rapid HIV testing. We found that none of the five articles met Drummond’s entire 10-item checklist (Table 2); we had no study that satisfied all ten criteria using the checklist. Other limitations of the checklists were the lack of a developed scoring algorithm. Using the CHEERS criteria, each ‘Yes’ response was weighted against the total number of criteria for an aggregate score.

Finally, we cannot exclude publication bias as almost all the economic evaluations showed the cost-effectiveness of rapid HIV testing.

## Conclusion

In conclusion, evidence exists from the United States that supports rapid HIV testing approaches compared to conventional HIV testing approaches. The evidence from this review is from a single low HIV prevalence high-income country. It does not specifically account for the difference in healthcare system characteristics and population contexts, making it difficult to generalize the evidence to other high-income, low HIV prevalence countries. The costs and outcomes associated with rapid HIV testing approaches suggest a cost-effective approach for population HIV screening, particularly among higher prevalence communities. However, there is inconsistent evidence of the use of rapid HIV testing approaches in lower prevalence settings. It would be of significant benefit to obtain estimates from other contexts and other countries besides the United States to account for the differences in healthcare system characteristics and enable more reliable generalization to these settings.

## Data Availability

The studies included in the review are publicly available.

## Abbreviations

CADTH: Canadian Agency for Drug Technologies and Health
CEA: Cost-Effectiveness Analysis
CEPAC: Cost-Effectiveness of Preventing AIDS Complications
ED: Emergency Department
HIV: Human Immunodeficiency Virus
QALY: Quality-Adjusted Life Years
PRISMA: Preferred Reporting Items for Systematic Reviews and Meta-Analyses
